# Navigating the treatment landscape in multiple myeloma: which combinations to use and when?

**DOI:** 10.1007/s00277-018-3546-8

**Published:** 2018-11-23

**Authors:** Hartmut Goldschmidt, John Ashcroft, Zsolt Szabo, Laurent Garderet

**Affiliations:** 1 Internal Medicine V and National Center for Tumor Diseases (NCT), University Clinic Heidelberg, 69120 Heidelberg, Germany; 2 Department of Haematology, Mid Yorkshire Hospitals NHS Trust, Wakefield, UK; 3 Clinical Development, Amgen (Europe) GmbH, Zug, Switzerland; 4 INSERM, UMR_S 938, Proliferation and Differentiation of Stem Cells, Paris, 75012 France; 5 AP-HP, Hôpital Saint Antoine, Département d’hématologie et de thérapie cellulaire, Sorbonne Université, Paris 6, Paris, France

**Keywords:** Multiple myeloma, Combination therapy, Sequential therapy, Treatment regimen

## Abstract

Multiple myeloma is one of the most common hematological malignancies, affecting mainly elderly patients. The treatment landscape for the management of this disease has evolved significantly over the past 15 years, and a vast array of therapeutics is now available, including immunomodulatory drugs, proteasome inhibitors, histone deacetylase inhibitors, and monoclonal antibodies. As a result, deciding which drugs to use and when, and whether these should be used in a particular order or combination, can be challenging. Although combination regimens are often associated with deeper responses and better long-term outcomes than monotherapy, and are becoming the standard of care, they may result in significant incremental toxicity; hence, a sequential approach may be more appropriate for some patients. In particular, treatment choices can vary depending on whether the patient has newly diagnosed multiple myeloma, is eligible for transplant, has relapsed and/or refractory multiple myeloma, or is considered to have high-risk disease. In this review, we discuss factors to be taken into account when making treatment decisions in each of these settings. We also briefly discuss possible therapeutic strategies involving agents that may become available in the future.

## Introduction

Multiple myeloma (MM) is a clonal plasma cell disorder that accounts for approximately 10% of hematological malignancies [1]. The disease has an estimated incidence of 4.5–6.0 per 100,000 people per year in Europe and primarily affects elderly patients, with a median age at diagnosis of 72 years [1]. Although MM remains an incurable disease, the development and introduction of therapies such as the immunomodulatory drugs thalidomide and lenalidomide and the proteasome inhibitor bortezomib have led to improved overall survival (OS) [2, 3]. Recent years have also seen the development and approval of numerous new treatments for patients with MM, including the second-generation proteasome inhibitors carfilzomib and ixazomib, of which carfilzomib demonstrated improved survival in a head-to-head study of carfilzomib plus dexamethasone versus bortezomib plus dexamethasone [4]. Other therapies with different mechanisms of action have emerged, including the immunomodulatory agent pomalidomide, the alkylating agent bendamustine, the histone deacetylase (HDAC) inhibitor panobinostat, and the monoclonal antibodies elotuzumab and daratumumab [5, 6]. Results from clinical trials suggest that the use of these agents may help to improve outcomes further [7–16].

Given the dramatic increase in therapeutic options available for patients with MM, one of the main challenges for physicians and funding bodies is deciding which agents to use and in which order and/or combination [17, 18]. Clonal heterogeneity is often observed in patients with MM, and it has been suggested that suboptimal treatment may lead to eradication of sensitive subclones while allowing resistant clones to expand [19]. As a result, combination therapy using agents from different drug classes with distinct and synergistic mechanisms of action is increasingly being utilized in an attempt to remove more subclonal groups, to reduce the risk of developing drug resistance and to induce a deeper response [5, 19]. For example, preclinical and clinical data suggest that a synergistic effect is observed when immunomodulatory drugs and proteasome inhibitors or monoclonal antibodies are used in combination [7, 10, 16, 20–22]. Immunomodulatory drugs stimulate natural killer cells and proteasome inhibitors may enhance natural killer cell-mediated cytotoxicity by reducing expression of host protein fragments on major histocompatibility complex (MHC) class I molecules [20]. In addition, monoclonal antibodies induce cell death via a number of mechanisms including antibody-dependent cell-mediated cytotoxicity (ADCC) and immunomodulatory drugs may enhance this anti-myeloma activity by activating the effector cells of ADCC [22]. However, when making treatment decisions, it is important to consider patient-related factors (i.e., age, comorbidities, and eligibility for autologous stem cell transplantation (ASCT)), disease-related factors (i.e., cytogenetics, disease burden, and aggressiveness of relapse in the relapsed/refractory disease setting) and previous therapies (i.e., number of previous therapy lines, response to previous therapies, and tolerability to previous therapies) [5, 17, 23]. Physicians also need to consider the balance between increasing the depth of response from a drug regimen and exposing patients to increased toxicity [24]. Although a deeper response is associated with better long-term outcomes [11, 14, 25, 26], the intensive multidrug therapy required to achieve this goal may result in significant treatment-related toxicity. Furthermore, the primary aim of treatment may differ between the newly diagnosed and relapsed/refractory settings, and this may influence the choice of drug regimen.

In this article, we review available therapies and provide guidance on the use of various treatment options in the newly diagnosed and relapsed/refractory settings. In addition, considerations for patients who are not eligible for ASCT are discussed, as well as for those who have high-risk disease.

## Management of patients with newly diagnosed multiple myeloma

### Transplant-eligible patients

**Fig. 1:**
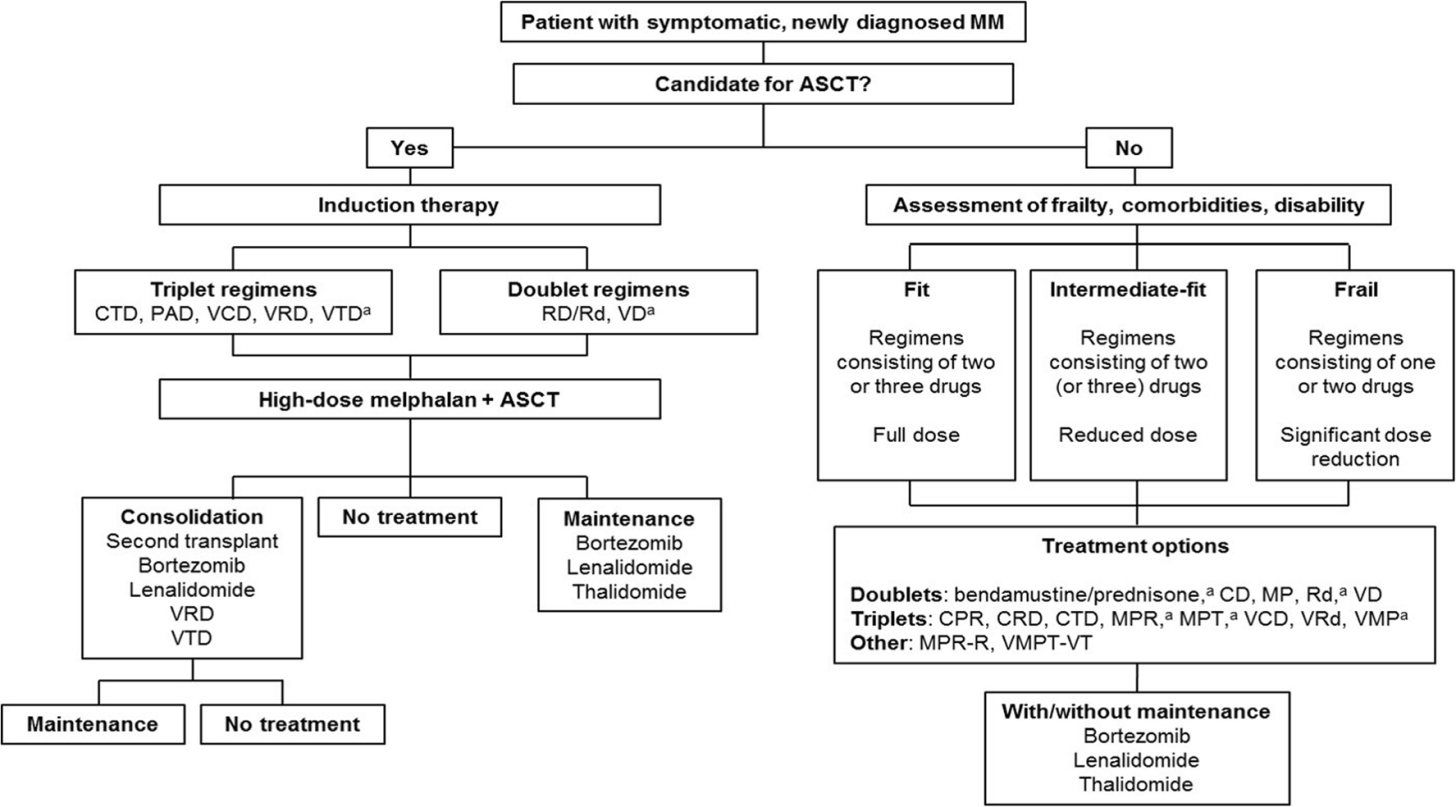
Treatment algorithm for patients with newly diagnosed multiple myeloma. *ASCT*, autologous stem cell transplantation; *CD*, cyclophosphamide and dexamethasone; *CPR*, cyclophosphamide, prednisone, and lenalidomide; *CTD*, cyclophosphamide, thalidomide, and dexamethasone; *MM*, multiple myeloma; *MP*, melphalan and prednisone; *MPR*, melphalan, prednisone, and lenalidomide; *MPR-R*, melphalan, prednisone, and lenalidomide, with lenalidomide maintenance; *MPT*, melphalan, prednisone, and thalidomide; *PAD*, bortezomib, doxorubicin, and dexamethasone; *Rd*, lenalidomide and low-dose dexamethasone; *RD*, lenalidomide and high-dose dexamethasone; *VCD*, bortezomib, cyclophosphamide, and dexamethasone; *VD*, bortezomib and dexamethasone; *VMP*, bortezomib, melphalan, and prednisone; *VMPT-VT*, bortezomib, melphalan, prednisone, and thalidomide, with bortezomib and thalidomide maintenance; *VRd*, lenalidomide, bortezomib, and low-dose dexamethasone; *VRD*, bortezomib, lenalidomide, and dexamethasone; *VTD*, bortezomib, thalidomide, and dexamethasone. ^a^Therapies approved by the European Medicines Agency

Treatment decisions in patients with newly diagnosed MM (NDMM) are usually made on the basis of age, performance status, and comorbidities. It is also important to take the patient’s preference into account [24]. In Europe, the standard of care for first-line therapy in patients up to 65 years of age and those considered to be in good clinical condition is induction therapy followed by high-dose melphalan and ASCT [1, 17]. The goals of induction therapy are to induce a deep response prior to ASCT, and this typically involves the use of combinations of two or three drugs in fit, transplant-eligible patients (Fig. 1) [1, 17, 24].

Triplet induction regimens are expected to result in deeper responses than doublet regimens, and several studies have demonstrated the efficacy of triplet combinations including the proteasome inhibitor bortezomib (Table 1) [27–34]. In patients with NDMM, induction therapy with bortezomib, thalidomide, and dexamethasone (VTD) or with vincristine, doxorubicin, and dexamethasone (VAD) has been shown to improve response rates compared with either thalidomide and dexamethasone (TD) or bortezomib and dexamethasone (VD) [27, 28, 30, 31]. A number of phase 3 trials compared the different available triplet regimens; bortezomib, doxorubicin, and dexamethasone (PAD) have demonstrated higher response rates and superior progression-free survival (PFS) and OS to VAD [32], and bortezomib, cyclophosphamide, and dexamethasone (VCD) have been shown to be non-inferior to PAD [33]. In a head-to-head comparison of VTD and VCD, VTD resulted in higher response rates than VCD [34]. Thus, three-drug combinations including at least bortezomib and dexamethasone are currently the standard of care before ASCT, with VTD and VCD as preferred regimens in Europe [1]. It is important to note that triplet regimens may be associated with toxicity issues. For example, the triplet VTD is associated with higher rates of peripheral neuropathy (PN) than the doublets TD and VD [27, 35]. Subcutaneous administration of bortezomib has been shown to be effective and to reduce the incidence of PN compared with intravenous administration [36]. Furthermore, the duration of treatment should be considered: the toxicity associated with a triplet regimen may be acceptable for an induction regimen, which is administered for a relatively short period. Although it is important to take toxicity into consideration when deciding on the most appropriate treatment for an individual, efficacy should be prioritized where possible, such as when making treatment decisions for fit patients who are eligible for ASCT.

**Table 1:** Key phase 3 studies of doublet and triplet regimens in transplant-eligible patients with newly diagnosed multiple myeloma

Study	Regimen	*N*	ORR (%)	≥VGPR (%)	CR (%)	Median PFS (months)	Median OS (months)
Cavo [27]	TD	238	79	28	5	–	–
	VTD	236	93	62	19	–	–
Harousseau [28]	VD	223	79	38	6	36.0	NR
	VAD	218	63	15	1	29.7	NR
Lokhorst [29]	TAD	268	–	37	3	34.0	73
	VAD	268	–	18	2	25.0	60
Moreau [30]	VD	99	81	36	12	30.0	–
	VTD	100	88	49	13	26.0	–
Rosinol [31]	TD	127	–	15^a^	14	8.2	–
	VTD	130	–	25^a^	35	56.2	–
Sonneveld [32]	VAD	414	–	14	2	28	NR
	PAD	413	–	42	7	35	NR
Mai [33]	VCD	251	–	37.0	8.4	–	–
	PAD	251	–	34.3	4.4	–	–
Moreau [34]	VCD	169	83	56	9	–	–
	VTD	169	92	66	13	–	–

ORR, VGPR, and CR refer to response to induction therapy

*CR*, complete response; *NR*, not reached; *ORR*, overall response rate; *OS*, overall survival; *PAD*, bortezomib, doxorubicin, and dexamethasone; *PFS*, progression-free survival; *Rd*, lenalidomide and low-dose dexamethasone; *TAD*, thalidomide, adriamycin, and dexamethasone; *TD*, thalidomide and dexamethasone; *VAD*, vincristine, doxorubicin, and dexamethasone; *VCD*, bortezomib, cyclophosphamide, and dexamethasone; *VD*, bortezomib and dexamethasone; *VGPR*, very good partial response; *VRd*, bortezomib, lenalidomide, and low-dose dexamethasone; *VTD*, bortezomib, thalidomide, and dexamethasone

^a^Proportion of patients with VGPR

While the efficacy of modern triplet combinations including the most recently approved drugs (such as carfilzomib, ixazomib, panobinostat, daratumumab, and elotuzumab) has been demonstrated in patients with relapsed and/or refractory MM(RRMM), they have not been extensively tested for first-line therapy. Nonetheless, positive results have been obtained in phase 2 trials of carfilzomib, lenalidomide, and low-dose dexamethasone (KRd) in patients with NDMM [37–39], as well as ixazomib, lenalidomide, and low-dose dexamethasone [40], and a phase 3 trial comparing KRd with bortezomib, lenalidomide, and low-dose dexamethasone (VRd) is in progress [41]. In addition, another clinical trial is investigating the use of therapy with either the triplets cyclophosphamide, lenalidomide, and dexamethasone (CRD) or cyclophosphamide, thalidomide, and dexamethasone (CTD) or a quadruple regimen, carfilzomib, cyclophosphamide, lenalidomide, and dexamethasone (CCRD) [42]. Findings from the randomized phase 3 Myeloma XI trial suggest that treatment with CCRD induces a deeper response than either of the triplet regimens [43, 44]; however, it should be noted that these data are preliminary and further research is needed to investigate fully the efficacy and safety of this approach. Another ongoing study in patients with NDMM is investigating induction therapy with VRD or VRD plus the monoclonal antibody elotuzumab, followed by maintenance therapy with lenalidomide with or without additional elotuzumab [45].

Although it was previously thought that potent combination therapy should be saved for use at relapse, it is now thought that its utilization earlier may increase the chances of obtaining a deep and durable response, resulting in improved outcomes. This is based on the hypothesis that potent treatment at an early stage may increase the likelihood of eradicating the majority of, or even all, subclones [46]. In addition, patients are more likely to have disease-related complications and comorbidities at later lines of treatment, which may impact on their ability to tolerate potent drug combinations, and so the use of the most effective combination treatments before these have developed may increase the likelihood of a sustained response [46]. Although further studies will be needed to determine the benefit of modern combination treatment regimens at early therapy lines, it is expected that these will help to improve responses and long-term outcomes.

In addition to induction therapy, consolidation and maintenance therapy may be given following ASCT. Consolidation therapy typically consists of a short period of intensive treatment with the aim of improving the depth of response after transplant [17, 47]. VTD is the predominant regimen used, but studies investigating the value of consolidation are limited [17, 47–49]. In contrast, maintenance therapy typically involves use of a more prolonged course of treatment with a lower-intensity regimen, with the aim of achieving long-term disease control [17, 47]. Maintenance therapy with thalidomide, lenalidomide, or bortezomib has been shown to have some benefit [32, 50–52]. Lenalidomide has been shown to improve OS compared with placebo or no maintenance therapy; a recent meta-analysis involving 1209 patients from three phase 3 randomized clinical trials of lenalidomide maintenance after ASCT demonstrated a significantly prolonged OS compared with controls [51]. Furthermore, the Myeloma XI study of more than 2000 patients with NDMM demonstrated that maintenance with lenalidomide was associated with a significantly longer median PFS compared with observation across all patient subgroups, including in those with high-risk disease [53]. In 2017, the use of lenalidomide maintenance therapy was approved for patients with NDMM following ASCT in Europe and the USA [54, 55]. In a head-to-head trial comparing bortezomib-based induction and maintenance therapies (PAD induction, bortezomib maintenance) versus VAD induction and thalidomide maintenance, the bortezomib group achieved superior PFS, an effect that was maintained for up to 96 months of follow-up; OS was similar with both treatments [32, 56]. Importantly, with prolonged bortezomib maintenance therapy for 96 months, there was no increased risk of second primary malignancies, which are an important complication for long-term survivors of MM [56]. A number of clinical trials to assess the use of newer agents (such as ixazomib, carfilzomib, elotuzumab, daratumumab, vorinostat, and panobinostat) for maintenance therapy are ongoing.

#### Summary

For transplant-eligible patients the goal is to achieve the deepest response and, if possible, a state of sustained minimal residual disease negativity. Consequently, use of a triplet regimen, such as VTD, VRd, or VCD, for induction prior to ASCT is recommended, providing toxicities allow. Owing to its potential to prolong PFS and OS, lenalidomide maintenance should be considered post-ASCT for all patients in whom it is tolerated. Additional clinical studies are needed to confirm the value of consolidation treatment after ASCT, as well as the use of newer therapies for maintenance [1].

### Transplant-ineligible patients

While using the most effective agents is the main strategy for newly diagnosed, transplant-eligible patients, this is not always appropriate for transplant-ineligible patients, who are usually older and may be considered less fit owing to comorbidities, disability, or disease burden. As a result, regimens that are suitable for transplant-eligible patients may be associated with toxicity issues that lead to early treatment discontinuation, resulting in low efficacy and poor quality of life in transplant-ineligible patients [57]. For example, although data are preliminary, the doublet VD has been shown to be as effective as the triplets bortezomib, melphalan, and prednisone (VMP) and VTD, and is associated with reduced toxicity in transplant-ineligible patients [35]. Therapy in these patients frequently focuses on controlling symptoms and preserving vital organ function, performance status, and quality of life [57].

A number of studies have investigated the efficacy of triplet and doublet regimens in patients not eligible for ASCT (Table 2) [35, 58–65], and there is some evidence to suggest that the use of a doublet may be more appropriate than a triplet [35, 62, 63]. For example, improved PFS and OS were demonstrated in a phase 3 study comparing lenalidomide and low-dose dexamethasone (Rd) with melphalan, prednisone, and thalidomide (MPT) [63]. Furthermore, a phase 3 study demonstrated that triplet lenalidomide-based regimens (melphalan, prednisone, and lenalidomide (MPR) and cyclophosphamide, prednisone, and lenalidomide (CPR)) were not associated with a significant difference in PFS compared with Rd in elderly patients with NDMM [62]. However, for some patients, sequential regimens may be suitable and the use of VMP and Rd administered in either a sequential or an alternating manner has been shown to be feasible, producing a similar outcome (in terms of 18-month PFS) to the trials of continuous regimens reported so far [66]. Furthermore, the use of reduced intensity bortezomib-based triplet regimens (VMP or VTP) followed by maintenance with a doublet regimen of VT or VP has been shown to be effective and more tolerable than higher intensity treatment in elderly patients with NDMM; notably, VMP was associated with fewer serious adverse events than VTP [59]. However, there is evidence to suggest that some patients may benefit from a triplet regimen; the phase 3 SWOG S0777 study demonstrated that induction therapy with VRd improved PFS and OS, compared with Rd, and had an acceptable risk-benefit profile in patients with NDMM without intent for immediate ASCT [64]. Interestingly, recent data from the phase 3 ALCYONE study in transplant-ineligible patients show that, compared with VMP, addition of daratumumab to VMP resulted in significantly higher rates of complete response and 18-month PFS. However, the quadruplet regimen was associated with a higher rate of grade 3 or 4 infections compared with the triplet regimen [65].

**Table 2:** Key phase 3 studies of doublet and triplet regimens in transplant-ineligible patients with newly diagnosed multiple myeloma

Study	Regimen	*N*	ORR (%)	VGPR (%)	CR (%)	Median PFS (months)	Median OS (months)
Palumbo [58]	MP	164	–	11	4	14.5	47.6
	MPT	167	–	29	16	21.8	45.0
Mateos [59]	VMP	130	80	–	20	34	NR
	VTP	130	81	–	28	25	NR
Niesvizky [35]	VD	168	73	–	3	14.7	49.8
	VTD	167	80	–	4	15.4	51.5
	VMP	167	70	–	4	17.3	53.1
Stewart [60]	MPT-T	154	64	20	5	21.0	52.6
	MPR-R	152	60	20	11	18.7	47.7
Hungria [61]	TD	18	69	19	13	21.5	54.6
	CTD	32	90	35	21	25.9	32.4
	MPT	32	68	25	14	24.1	42.0
Magarotto [62]	Rd	212	74	31	3	21.0	NR
	CPR	220	68	20	1	20.0	NR
	MPR	211	71	23	3	24.0	NR
Hulin [63]	Cont. Rd	535	81	27	21	26.0	58.9
	Rd18	541	79	27	20	21.0	56.7
	MPT	547	67	18	12	21.9	48.5
Durie [64]	Rd	214	72	23	8	30.0	64.0
	VRd	216	82	28	16	43.0	75.0
Mateos [65]	VMP	356	74	25	24	18.1	NR
	DVMP	350	91	29	43	NR	NR

*Cont. Rd*, continuous lenalidomide and low-dose dexamethasone; *CPR*, cyclophosphamide, prednisone, and lenalidomide; *CR*, complete response; *CTD*, cyclophosphamide, thalidomide, and dexamethasone; *DVMP*, daratumumab, bortezomib, melphalan, and prednisone; *MP*, melphalan and prednisone; *MPR*, melphalan, prednisone, and lenalidomide; *MPR-R*, melphalan, prednisone, and lenalidomide with lenalidomide maintenance; *MPT*, melphalan, prednisone, and thalidomide; *MPT-T*, MPT with thalidomide maintenance; *NR*, not reached; *ORR*, overall response rate; *OS*, overall survival; *PFS*, progression-free survival; *Rd*, lenalidomide and low-dose dexamethasone; *Rd18*, lenalidomide and low-dose dexamethasone for 72 weeks (18 cycles); *TD*, thalidomide and dexamethasone; *VD*, bortezomib and dexamethasone; *VGPR*, very good partial response; *VMP*, bortezomib, melphalan, and prednisone; *VTD*, bortezomib, thalidomide, and dexamethasone; *VTP*, bortezomib, thalidomide, and prednisone

When making treatment decisions for transplant-ineligible patients, it is important that factors such as patient age, comorbidities, degree of frailty, and patient preference are taken into account [17, 57, 67, 68]. The International Myeloma Working Group (IMWG) recently developed a scoring system based on age, comorbidities, and cognitive and physical condition to classify patients in “fit,” “intermediate-fit”, and “frail” groups [67, 68]. Management strategies can then be tailored accordingly (Fig. 1). For example, regimens consisting of two or three drugs at full dose may be appropriate for patients defined as fit, while those defined as intermediate-fit may be treated with two (or three) drugs at a reduced dose, and those defined as frail with one or two drugs at a significantly reduced dose [17, 57]. In particular, CTD, MPT, CRD, Rd, VRd, or VMP may be suitable for fit or intermediate-fit patients [61, 69–71], while MP or cyclophosphamide and dexamethasone (CD) may be more appropriate for intermediate-fit or frail patients.

#### Summary

Many transplant-ineligible patients, and particularly those who are frail, are unlikely to tolerate aggressive combinations. Instead, a more conservative approach employing the use of reduced intensity combination or sequential treatments, that takes into consideration possible toxicity issues and likely tolerability, may be more appropriate in this group of patients. However, it is important to assess the fitness of transplant-ineligible patients because those who are ‘fit’ may be able to benefit from two- or three-drug regimens used at full dose.

## Management of patients with relapsed/refractory multiple myeloma

**Fig. 2:**
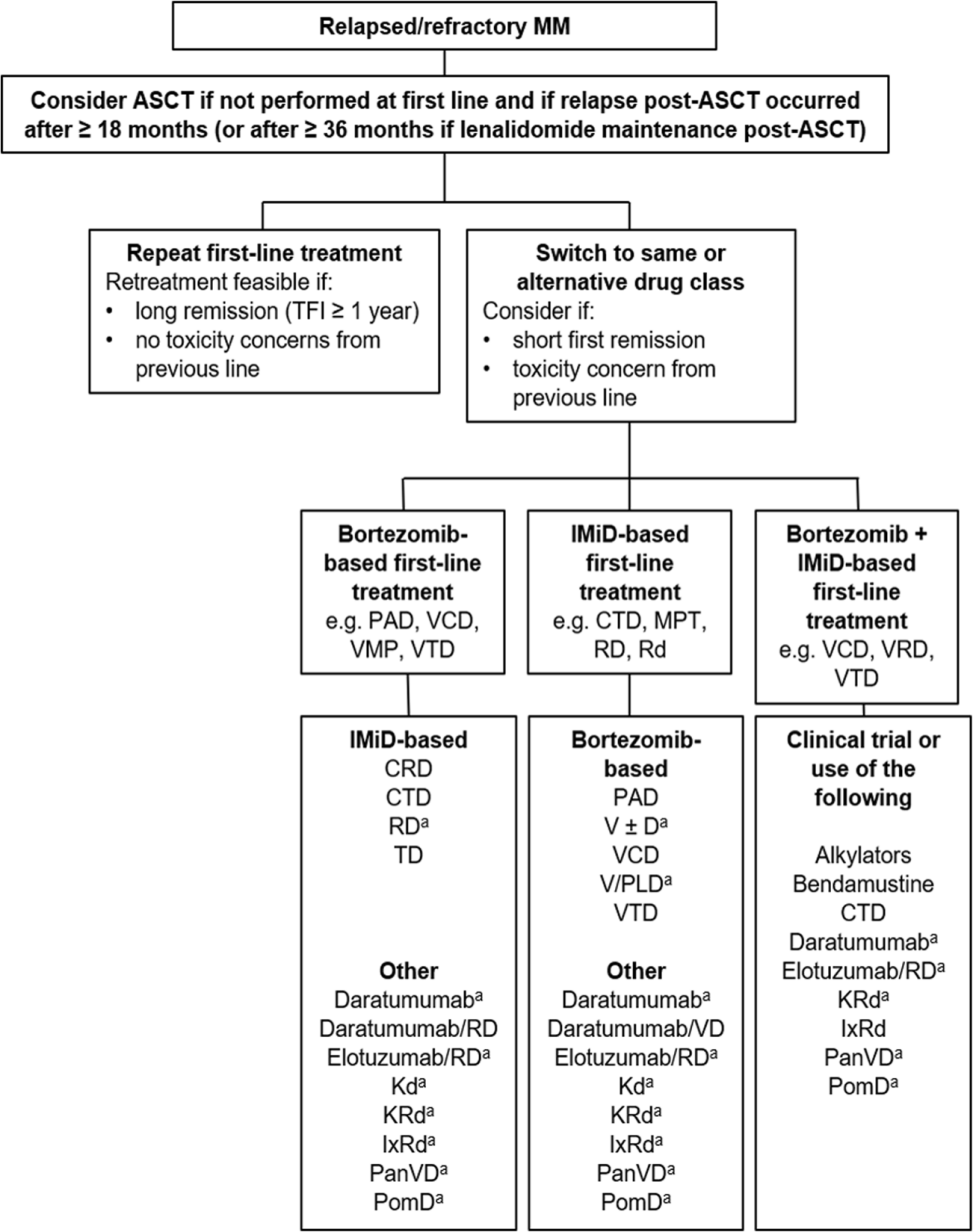
Treatment algorithm for patients with relapsed/refractory multiple myeloma. *ASCT*, autologous stem cell transplantation; *CRD*, cyclophosphamide, lenalidomide, and dexamethasone; *CTD*, cyclophosphamide, thalidomide, and dexamethasone; *IMiD*, immunomodulatory drug; *IxRd*, ixazomib, lenalidomide, and low-dose dexamethasone; *Kd*, carfilzomib and low-dose dexamethasone; *KRd*, carfilzomib, lenalidomide, and low-dose dexamethasone; *MM*, multiple myeloma; *MPT*, melphalan, prednisone, and thalidomide; *PAD*, bortezomib, doxorubicin, and dexamethasone; *PanVD*, panobinostat, bortezomib, and dexamethasone; *PomD*, pomalidomide and dexamethasone; *Rd*, lenalidomide and low-dose dexamethasone; *RD*, lenalidomide and high-dose dexamethasone; *TD*, thalidomide and dexamethasone; *TFI*, treatment-free interval; *VCD*, bortezomib, cyclophosphamide, and dexamethasone; *V* ± *D*, bortezomib with or without dexamethasone; *VMP*, bortezomib, melphalan, and prednisone; *V/PLD*, bortezomib and pegylated liposomal doxorubicin; *VRD*, bortezomib, lenalidomide, and dexamethasone; *VTD*, bortezomib, thalidomide, and dexamethasone. ^a^Therapies approved by the European Medicines Agency

Relapse of MM is considered to be almost inevitable and the management of patients with RRMM requires an individualized approach. This should take into account the patient’s age, fitness, comorbidities, treatment history (including both depth and duration of the response, as well as treatment toxicities), and aggressiveness of the relapse, as well as the expectations of the patient and his or her quality of life [17, 72, 73]. Treatment options include re-treating with an agent used previously, switching to a different agent in the same drug class, or switching to an agent in a different drug class (Fig. 2). ASCT may be considered as a salvage option if patients are transplant-eligible and have either never received an ASCT or had a previous ASCT with a long response duration [73–77]. Patients should also be considered for participation in clinical trials, if available [17, 72, 73, 78].

In general, doublet or triplet regimens are used in RRMM (Table 3) [7–10, 14–16, 79, 80], with the specific treatment choice dependent upon the expected efficacy, toxicity, and possible cost considerations, as well as the fitness of the patient [17, 72, 78]. Bortezomib, thalidomide, and lenalidomide were frequently used for the treatment of RRMM [17, 72, 73]. For example, combinations such as VTD, VRD, VCD, and bortezomib with doxorubicin and dexamethasone (PAD) may still be considered; in particular, the triplet VTD has been shown to be superior to TD in patients with relapsed disease post ASCT [17, 79]. However, the presence of comorbidities such as PN may mean that changes are made to the dose or schedule, or necessitate use of an alternative drug class [72, 78, 81]. Rd is also an effective option [72, 82, 83] and TD may be appropriate, especially if patients are thalidomide-naïve or are not eligible for bortezomib or lenalidomide-based treatment [72].

**Table 3:** Key phase 3 studies of doublet and triplet regimens in patients with relapsed and/or refractory multiple myeloma

Study	Regimen	*N*	ORR (%)	VGPR (%)	CR (%)	Median PFS (months)	Median OS (months)
Garderet [79]	TD	134	72	14	13	13.6	–
	VTD	135	87	11	28	18.3	–
San-Miguel [14]	VD	381	55	–	6	8.1	30.4
	PanVD	387	61	–	11	12.0	33.6
Baz [80]	PomD	36	39	–	–	4.4	10.5
	PCD	34	65	–	–	9.2	16.4
Stewart [7]	Rd	396	67	40	5	17.6	NR
	KRd	396	87	70	18	26.3	NR
Lonial [9]	Rd	325	66	21	7	14.9	NR
	Rd + elotuzumab	321	79	28	4	19.4	NR
Moreau [10]	Rd	362	72	32	7	14.7	NR
	IxRd	360	78	36	12	20.6	NR
Palumbo [15]	Vd	247	63	20	7	7.2	–
	Vd + daratumumab	251	83	40	15	NR	–
Dimopoulos [4, 8]	Kd	464	77	42	13	18.7	47.6
	Vd	465	63	22	6	9.4	40.0
Dimopoulos [16]	Rd	283	76	25	19	18.4	–
	Rd + daratumumab	286	93	33	43	NR	–

*CR*, complete response; *IxRd*, ixazomib, lenalidomide, and low-dose dexamethasone; *Kd*, carfilzomib and low-dose dexamethasone; *KRd*, carfilzomib, lenalidomide, and low-dose dexamethasone; *NR*, not reached; *ORR*, overall response rate; *OS*, overall survival; *PanVD*, panobinostat, bortezomib, and dexamethasone; *PCD*, pomalidomide, cyclophosphamide, and dexamethasone; *PFS*, progression-free survival; *PomD*, pomalidomide and dexamethasone; *Rd*, lenalidomide and low-dose dexamethasone; *TD*, thalidomide and dexamethasone; *Vd*, bortezomib and low-dose dexamethasone; *VD*, bortezomib and dexamethasone; *VGPR*, very good partial response; *VTD*, bortezomib, thalidomide, and dexamethasone

The development of novel agents for the treatment of MM has significantly increased the range of possible treatment combinations and these may result in even better outcomes for patients with RRMM. KRd has been shown to have a significant impact on PFS and OS, with a favorable risk–benefit profile and improved health-related quality of life compared with Rd [7, 84]. The benefit of this regimen in terms of PFS was observed in all patients, including those who had previously received treatment with bortezomib or lenalidomide or who had high-risk cytogenetics. The combination of carfilzomib with low-dose dexamethasone (Kd) has also been shown to result in improved PFS and OS compared with bortezomib combined with low-dose dexamethasone (Vd) [4, 8]. PN has been shown to be less frequent in patients treated with carfilzomib than in those receiving bortezomib, and addition of carfilzomib to Rd did not add PN toxicity, so carfilzomib regimens may be a suitable option for patients including those with existing or anticipated neuropathy [8, 78].

Another second-generation proteasome inhibitor, ixazomib, has been developed, and the combination of ixazomib with lenalidomide and low-dose dexamethasone has been shown to result in improved PFS compared with placebo, lenalidomide, and low-dose dexamethasone in patients with RRMM [10]. Ixazomib is available in the USA and received approval for use in Europe in December 2016 [10, 85]. In addition, the availability of the immunomodulatory agent pomalidomide and the HDAC inhibitor panobinostat has opened up the possibility of using doublet or triplet regimens in patients with advanced disease [11, 12, 14]. In particular, pomalidomide in combination with dexamethasone is suitable for use in patients who have received at least two previous treatment regimens and have demonstrated disease progression [12, 86]. Studies investigating the use of pomalidomide, bortezomib, and dexamethasone (PVd), pomalidomide, cyclophosphamide, and prednisone (PCP), pomalidomide, cyclophosphamide, and dexamethasone (PCD) and pomalidomide, carfilzomib, and dexamethasone (PKD), also suggest that these regimens may be effective in RRMM [11, 80, 87, 88]. Additionally, although not currently approved in the relapsed setting, the alkylating agent bendamustine in combination with thalidomide and dexamethasone has been shown to be a viable salvage therapy for patients with relapsed disease who are refractory to bortezomib and lenalidomide [13].

The use of monoclonal antibodies is well established for the treatment of other cancers and although these agents have only recently become available for the treatment of RRMM, they are already showing promise. The anti-CD38 monoclonal antibody daratumumab has been shown to be effective in patients with RRMM in a number of studies, either as monotherapy or in combination with lenalidomide and dexamethasone [89, 90]. In addition, results from a phase 3 trial of daratumumab with bortezomib and dexamethasone (CASTOR) demonstrated a significant improvement in PFS compared with bortezomib and dexamethasone alone [15]; PFS was also found to be improved in patients treated with daratumumab, lenalidomide, and dexamethasone versus those who received lenalidomide and dexamethasone only (POLLUX) [16]. In addition, the anti-SLAM-7 antibody elotuzumab combined with lenalidomide and dexamethasone was associated with improved PFS in patients with RRMM compared with lenalidomide and dexamethasone alone [9]. Results from a phase 2 study also indicate improved PFS in patients treated with elotuzumab, bortezomib, and dexamethasone versus those treated with bortezomib and dexamethasone alone [91]. These findings suggest that monoclonal antibodies will have an important role in the future treatment of patients with RRMM, and several ongoing trials are assessing their value when combined with other therapeutic agents [92–95].

When making treatment decisions, the prognosis may influence the therapeutic strategy chosen. For example, recent recommendations from the IMWG suggest that patients with a poor prognosis at relapse are treated with a triplet or quadruplet regimen, until disease progression [73]. In this setting, novel treatments may be more appropriate than bortezomib or alkylating agents, because these agents are suitable for use until disease progression [54, 85, 96, 97]. In contrast, it is recommended that patients with indolent disease characteristics are treated with one- or two-drug regimens and treatment-free intervals may be appropriate for these individuals [73]. In addition, existing comorbidities may make it necessary to consider dose adjustments, for example reduced doses of ixazomib and lenalidomide are recommended for patients with severe renal impairment [54, 85].

The response to previous therapies must also be taken into account when making treatment decisions. Re-treatment with a drug used previously should be feasible, provided that a clinically meaningful response was achieved, the previous response lasted for at least 12 months, and treatment was associated with acceptable toxicity [17, 72, 78]. In patients who demonstrate disease progression while on therapy, or who had only a short response, switching to a different drug class should be considered [73]. For example, if lenalidomide was used as first-line treatment, bortezomib could be used at relapse. Alternatively, it may be possible to use a second-generation agent in the same drug class as the treatment used at first line. For example, carfilzomib is structurally and mechanistically distinct from bortezomib and has been shown to be effective in patients who previously received bortezomib [7, 8]. In addition, studies have demonstrated that pomalidomide is effective in patients for whom lenalidomide has failed [98], as well as in those who are refractory to both lenalidomide and bortezomib [12, 99]. Given the number of novel agents now available in both existing and new drug classes, it should be possible for patients to receive treatment at relapse with an agent that they are not resistant to, even if they receive a combination treatment initially. However, long-term data on the use of the newest agents following different initial treatment combinations are limited and so further studies will be needed to determine whether particular sequences of drug regimens are associated with improved responses and long-term outcomes.

### Summary

In the relapse setting, triplet regimens including lenalidomide, dexamethasone, and either a monoclonal antibody or a proteasome inhibitor are recommended, although this may depend on the therapies employed at first line. If a bortezomib-based regimen was used at first line, a lenalidomide-based regimen can be used at relapse and vice versa. Regimens employing the next-generation immunomodulatory drug pomalidomide are currently under investigation and are likely to prove useful for patients with RRMM [100, 101].

## Considerations for patients with standard-risk versus high-risk disease

Although response to treatment and survival of patients with MM is highly variable, there are certain prognostic factors that can be used to predict the clinical course of the disease [1]. Patients can be stratified into high-risk and standard-risk disease groups according to the presence or absence of various disease characteristics such as cytogenetic abnormalities, elevated serum β2-microglobulin levels, and elevated serum lactate dehydrogenase levels (Table 4) [73, 102–104]. Treatment decisions can then be made on the basis of these risk classifications.

**Table 4:** High-risk disease characteristics in multiple myeloma [73, 102–104]

R-ISS stage	• R-ISS III - serum β2-microglobulin level > 5.5 mg/L and either high-risk chromosomal abnormalities [del(17p) and/or t(4;14) and/or t(14;16)] or high serum LDH (> upper limit of normal)
Host characteristics	• Advanced age
	• Low performance status
	• Increased comorbidities
Disease characteristics	• Presence of extramedullary disease
	• Aggressive clinical features, including: - Rapid onset of clinical symptoms - Extensive disease at relapse (based on laboratory, pathology, or radiographic findings) - Disease-associated organ dysfunction at relapse (including renal failure, hypercalcemia, cytopenias, or bone event such as fracture)
	• Circulating plasma cells
	• Reduced polyclonal bone marrow plasma cells
	• High serum free light chain

*LDH*, lactate dehydrogenase; *R-ISS*, revised International Staging System

In general, patients with high-risk disease (including high-risk cytogenetics and/or high tumor load) should be treated with a triplet regimen, if possible. Trials studying the use of thalidomide during induction therapy in transplant-eligible patients with NDMM indicate that this therapy does not overcome the adverse prognosis associated with high-risk cytogenetics [26, 31, 103, 105]. However, patients with t(4;14) may benefit from a proteasome inhibitor-based treatment, and the combination of a proteasome inhibitor with lenalidomide and dexamethasone has been recommended by the IMWG for newly diagnosed transplant-eligible patients with high-risk cytogenetics [103, 106]. Results from a recent post hoc analysis suggest that cytogenetic risk should also be taken into account in the context of sequential therapy. While PFS at second line (PFS2) was not influenced by treatment sequence in patients with standard-risk cytogenetics, PFS2 was reduced in individuals with high-risk cytogenetics who received lenalidomide upfront followed by bortezomib at first relapse, compared with those who received the same treatment at relapse or who received bortezomib upfront [107]. In addition, double high-dose therapy/ASCT combined with bortezomib may improve PFS in patients with high-risk cytogenetics (t(4;14) or del(17p)) [103].

In patients with RRMM and high-risk disease, doublet therapy consisting of pomalidomide plus low-dose dexamethasone may be a suitable option [73, 108]; in particular, this therapy option has been shown to be active in patients with del(17p) [109]. The phase 3 trials of KRd and Kd in patients with RRMM suggest that carfilzomib is also effective in patients with high-risk cytogenetics [7, 8, 110]. Similarly, subgroup analysis of the phase 3 TOURMALINE-MM1 trial of ixazomib plus Rd suggest that this combination is effective in patients with high-risk cytogenetics [40]. Additionally, recent subgroup analyses of the phase 3 POLLUX and CASTOR trials suggest that, compared with control treatment, daratumumab improves response rates and PFS in patients with high-risk disease and in those with standard-risk disease [111, 112]. One other option for high-risk patients with relapsed MM is allogeneic stem cell transplantation. However, this is suitable only for a subset of patients who are young and who have an available human leukocyte antigen-matched donor, chemotherapy-sensitive disease, and an excellent performance status, and it should ideally be performed in the context of a clinical trial [73].

### Summary

The treatment of patients with high-risk disease with a triplet regimen including a proteasome inhibitor and an immunomodulatory drug is recommended. A triplet regimen, perhaps including the novel immunomodulatory drug pomalidomide or a proteasome inhibitor such as carfilzomib, may be an option for patients with RRMM and high-risk disease.

## Future treatment strategies

Multiple myeloma should not be considered to be one disease, but rather a mix of different disease entities that further interact with individual patient characteristics [102]. Risk stratification is crucial to identify patients with a high risk of early relapse in order to adapt treatment regimens accordingly; however, further work is required to develop tools that take into account the broad spectrum of factors that define risk, both in the front-line and relapsed settings [102, 113]. Beyond risk stratification, the advent of various high-throughput technologies in myeloma cell genotyping and phenotyping are bringing personalized myeloma therapy ever closer. Gene expression profiling of malignant plasma cells is a promising method for prognostication and may inform treatment choices [114–116]. For example, the presence of *NRAS* mutations has been shown to be associated with poor response to bortezomib [117]. Conversely, mutations in *IRF4* are associated with favorable outcomes following immunomodulatory agent therapy [118]. Finally, the identification of novel mutations may lead to the development of new targeted therapies in myeloma [118]. For example, overexpression of BCL-2 has been implicated in the growth of t(11;14) myeloma cells and preliminary results from a phase 1 study suggest that the BCL-2 inhibitor venetoclax may be effective in treating patients with t(11;14) [119].

Given the array of therapeutic options available and the efficacy of triplet regimens, it might be expected that use of quadruplet regimens would result in even better outcomes. The efficacy and safety of quadruplet regimens have been investigated in a limited number of studies; although preliminary data suggest that the quadruplet CCRD is effective [43], studies of other quadruplet regimens have reported toxicity issues [120]. Further studies will be needed to assess the value of these regimens. A number of phase 3 studies assessing the value of quadruplet regimens including a monoclonal antibody are ongoing [121, 122].

Other new therapeutic agents are under investigation, including novel proteasome inhibitors (oprozomib and marizomib), HDAC inhibitors (romidepsin, vorinostat, ricolinostat), monoclonal antibodies (SAR650984, MOR202, isatuximab, ipilimumab), and small-molecule inhibitors (vemurafenib, venetoclax, CPI-0610, LGH447, dinaciclib, selinexor, ibrutinib, and filanesib) [6, 23, 95, 123]. The efficacy of these remains to be fully tested; however, they should help to expand the range of therapeutic options available. This is particularly important because the use of combination therapies at first line increases the risk of developing resistance to multiple classes of drug, necessitating the use of different agents at later lines. In addition, the use of existing therapies has already been shown to be associated with high costs [124], and it is likely that novel agents will increase these further, placing a significant burden on healthcare providers and funding bodies. As more novel agents emerge, cost-effectiveness analyses will be needed to establish the value of adopting combination regimens. Nonetheless, it seems probable that the development of new treatments is likely to result in improvements in the long-term management of patients with MM and raises the possibility that in the future it may be possible to cure the disease, particularly in patients who are able to tolerate combination therapy with a range of different agents.

## Conclusions

The treatment landscape for MM has evolved significantly over the past decade, and several therapeutic options are now available. In particular, the development and availability of monoclonal antibodies may well lead to a treatment paradigm shift whereby the use of a monoclonal antibody in combination with a doublet or triplet regimen may be suitable for treatment of the disease. Of course, the heterogeneity of MM means that an individualized approach is still required when making treatment decisions. This should involve risk stratification and the assessment of the patient’s frailty, disabilities, and comorbidities and, in the RRMM setting, consideration of previous treatment history and response.

The availability of novel agents makes combinations of drugs from different classes possible, and the latest results from clinical studies suggest that the efficacy benefits of treatment combinations involving these agents are likely to outweigh the risk of patients developing multi-drug resistance. However, it remains important for physicians to consider the aims of treatment carefully, and to ensure that there is an appropriate balance between response and toxicity. There is also a need to investigate novel treatment combinations and sequences further, with the aim of achieving greater responses while minimizing treatment-related toxicity, as well as the potential benefits of treating patients with high-risk smoldering MM. Additional work in these areas should ultimately lead to improved treatment regimens and outcomes for patients with MM.
